# SARS-CoV-2 Catalonia contact tracing program: evaluation of key performance indicators

**DOI:** 10.1186/s12889-022-13695-8

**Published:** 2022-07-20

**Authors:** Mercè Herrero, Pilar Ciruela, Meritxell Mallafré-Larrosa, Sergi Mendoza, Glòria Patsi-Bosch, Èrica Martínez-Solanas, Jacobo Mendioroz, Mireia Jané, Epidemiological Surveillance Network of Catalonia, Gloria Carmona Parcerisa, Ana Isabel Martinez Mateo, Sandra Pequeño Saco, Agata Raquel Leon Garcia, Elisabet Domenech, Carles Pericas Escalé, Irene Barrabeig Fabregat, Ariadna Rovira Faixa, Mònica Carol Torrades, Victor Guadalupe Fernandez, Nuria Bes Montalat, M. Nuria Follia Alsina, Joaquim Ferras Prats, Sofía Minguell Verges, Gloria Ferrús Serra, Pere Godoy Garcia, Miquel Alseda Graells, Ignacio Parron Bernabe, Anna Cirera Viladot, Cristina Rius Gibert, Patricia García de Olalla Rizo, Glòria Pérez Albarracín, Maria-Rosa Sala Farre, Laura Clotet Romero, Mercè Herrero Garcia, Pilar Ciruela Navas, Meritxell Mallafré-Larrosa, Sergi Mendoza Funes, Glòria Patsi-Bosch, Èrica Martínez-Solanas, Jacobo Mendioroz Peña, Mireia Jané Checa

**Affiliations:** 1 Sub-Directorate General of Surveillance and Response to Public Health Emergencies, Public Health Agency of Catalonia, Generalitat of Catalonia, 08005 Barcelona, Spain; 2 CIBER Epidemiologia y Salud Pública (CIBERESP), Instituto Salud Carlos III, 28029 Madrid, Spain; 3 Research Support Unit of Central Catalonia, University Institute for Research in Primary Health Care Jordi Gol i Gurina, 08272 Sant Fruitós de Bages, Spain; 4 Public Health, Barcelona University, Barcelona, Spain

**Keywords:** SARS-CoV-2, COVID-19, Contact tracing, Program evaluation, Key performance indicator

## Abstract

**Background:**

Guidance on SARS-CoV-2 contact tracing indicators have been recently revised by international public health agencies. The aim of the study is to describe and analyse contact tracing indicators based on Catalonia’s (Spain) real data and proposing to update them according to recommendations.

**Methods:**

Retrospective cohort analysis including Catalonia’s contact tracing dataset from 20 May until 31 December 2020. Descriptive statistics are performed including sociodemographic stratification by age, and differences are assessed over the study period.

**Results:**

We analysed 923,072 contacts from 301,522 SARS-CoV-2 cases with identified contacts (67.1% contact tracing coverage). The average number of contacts per case was 4.6 (median 3, range 1–243). A total of 403,377 contacts accepted follow-up through three phone calls over a 14-day quarantine period (84.5% of contacts requiring follow-up). The percentage of new cases declared as contacts 14 days prior to diagnosis evolved from 33.9% in May to 57.9% in November. All indicators significantly improved towards the target over time (*p* < 0.05 for all four indicators).

**Conclusions:**

Catalonia’s SARS-CoV-2 contact tracing indicators improved over time despite challenging context. The critical revision of the indicator’s framework aims to provide essential information in control policies, new indicators proposed will improve system delay’s follow-up**.** The study provides information on COVID-19 indicators framework experience from country’s real data, allowing to improve monitoring tools in 2021–2022. With the SARS-CoV-2 pandemic being so harmful to health systems and globally, is important to analyse and share contact tracing data with the scientific community.

**Supplementary Information:**

The online version contains supplementary material available at 10.1186/s12889-022-13695-8.

## Background

To date, the coronavirus disease (COVID-19) pandemic represents the biggest and most pressing global public health challenge, with more than 100 million people infected and over 2.1 million people who lost their lives as of December 2020 [1]. In the absence of a curative treatment and with limited percentage of worldwide population vaccinated mostly in high-income countries, a diagnostic testing, contact tracing, and supported isolation strategy (TTSI) is considered, as key measures of epidemiological surveillance to mitigate such a highly infectious and rapid spread disease recognizing both its long incubation period and relatively short disease course [2, 3]. Contact tracing (CT) is the process of identifying, assessing and managing people who have been exposed to a disease through a 14-day follow-up from the last point of exposure with the aim to reduce the time from symptom onset to isolation and subsequently prevent onward transmission breaking the chains of transmission [4, 5].

**Fig. 1:**
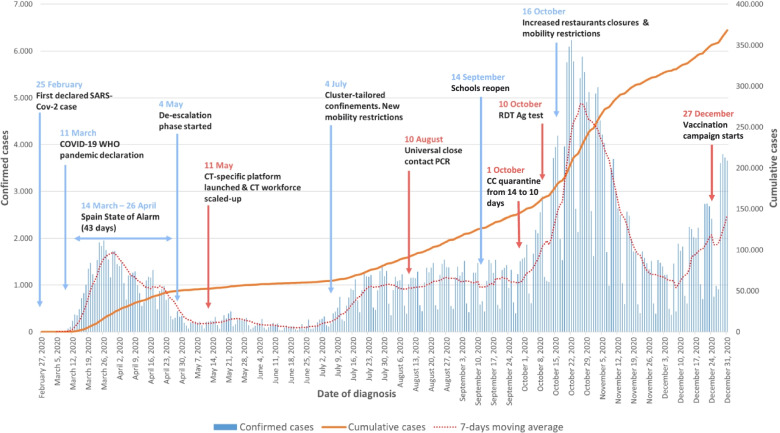
Temporal evolution of SARS-CoV-2 cases and accumulated incidence, main events and strategies. Catalonia, May–December 2020. *Red events more related with the contact tracing program

The first wave of COVID-19 pandemic spread rapidly in Spain, one of the most affected countries in Europe, together with Italy [6]. The first imported case in Spain was detected in the Canary Islands on January 31, 2020 [7]. After unsuccessful efforts to contain the infection and a clear situation of community transmission, on March 14, the Spanish government declared a state of alarm encompassing a number of restrictions in all its autonomous communities [8], notably entailing a strict lockdown. The strategy led to an inflection of the epidemic curve, as 6 days later the cases began to decrease [7], and the lockdown lasted up until May 11, 2020. A second wave took place early July and a third wave in October following summer time and reopening of the schools (Fig. 1).

Catalonia, one of the 17 regions of Spain with a population of 7.7 million [9], was hard hit over the first wave, having the basic reproductive number (R0) soared up to 4.6 during the first 2 weeks of March before lockdown measures [10] while in Spain was 5.89 (95% Confidence Interval (CI): 5.46–7.09) for the same period [11]. The first severe acute respiratory syndrome coronavirus 2 (SARS-CoV-2) confirmed case in Catalonia was on February 25, 2020, in a young woman who had been travelling Northern Italy [12], after a hard containment phase being implemented by the Epidemiological Surveillance Network of Catalonia (XVEC) between January and March 2020. From February 25 up to December 31, 2020, 301,522 cases were notified at the MediatorCovidContacts (MCC) platform [13, 14]. A national seroprevalence study conducted between April and May, 2020, reported an overall 5.0% (95% CI 4.7–5.4) presence of SARS-CoV-2 antibodies among the Spanish population, being greater in Catalonia and specially in the province of Barcelona with a 7.0% (95% CI 5,7 - 8,6) [15].

On March 14, 2020, the Catalan government put in place a strong public health response to effectively control the pandemic (Fig. 1 displays the strategy highlighted over the study period). It included a TTSI plan to maintain the gradual decrease of cases over the de-escalation phase [16], for which the XVEC was appointed responsible. This network was created in 2015 and links healthcare professionals and the epidemiological surveillance services intervening in Catalonia at different territorial levels. Before the pandemic, CT was already conducted by the XVEC for several disease monitoring programs, such as tuberculosis, measles, meningococcal disease or diphtheria disease, among others [17]. COVID-19 put a tremendous pressure on the existing public health system and healthcare providers, which had to adapt to the evolving pandemic increasing capacity at all levels. A call center started to run on 20 May, 2020, to trace and follow-up all SARS-CoV-2 cases’ close contacts (CC) as part of the support provided by the XVEC and similarly to the system carried out in other countries [18, 19]. Certain vulnerable populations were specifically targeted, such as nursery homes as well as those areas with high incidence [20].

CT programs are essential to contribute in preventing onward transmission in the community [4]. A set of CT indicators were used in Catalonia since the beginning of the pandemic based on international public health targets. As the pandemic evolved, revising the indicators is an evaluation exercise of the programme. The study describes and analyses the indicators used in the catalan CT program and, to our knowledge, constitutes the first country experience providing a systematic framework based on real country data, and highlights how to guide a COVID-19 control strategy according to CT performance.

## Methods

### Study design and population

We conducted a retrospective-ascertained study of CC from confirmed SARS-CoV-2 cases managed in Catalonia between 20 May and 31 December, 2020. Contacts were included from 48 hours prior to the first diagnosed case on 20 May, and followed up until 14 days after the last exposure to the index case, being January 14, 2021 the last follow-up date, according to our protocol [21].

This study followed the STROBE reporting guideline and all methods in the study were carried out in accordance with the Helsinki guidelines and declaration or any other relevant guidelines [22, 23]. Information was collected by case interviewers and contact tracers at all surveillance system levels according to the COVID-19 protocol from the Public Health Agency of Catalonia (PHAC). The protocol follows regular updates according to the pandemic control strategies changes [16, 20, 21, 24].

### Ascertainment of cases

A confirmed case met the criteria of COVID-19 notification in Catalonia if tested positive by polymerase chain reaction (PCR) tests, being the result stored in the Taga19 platform. Case detailed information was collected as part of the “Case epidemiological interview” included in the COVID-19 protocol [21], which contain demographic and clinical data. The case’s electronic card was transferred from Taga19 platform (from cases) to MCC platform (from contacts).

### Contact tracing program for COVID-19

CT in our protocol, follows the Centers for Diseases Control and Prevention procedure: “Contact Tracing refers to the process of notifying contacts of exposure, addressing questions and concerns, referring for SARS-CoV-2 testing, encouraging self-quarantine, monitoring of symptoms, and assessing the need for additional supportive services during the quarantine period (14 days from last exposure)” [25]. During case investigation we ask for CC and exposure setting, since 48 h prior to the date of symptom onset (and could be extended to up to 14 days before in case of local outbreak declaration) and ended at the date of case isolation. For asymptomatic confirmed cases, the period started 48 hours prior to the date of microbiological sample collection.

The definition of a CC was a person who did not wear appropriate personal protection equipment, while having face-to-face contact with a confirmed case for more than 15 min (throughout 24 h) within less than a 2-m distance during the investigation period.

All CC were notified through a telephonic call in which 14-day quarantine instructions were provided, starting from their last exposure to the index case and ending with at least 72 hours without symptoms. The process of “verification” consists in the capability to get in touch with a contact through phone at the first call. In this initial call, information on the CC prior exposure to a confirmed case, settings of exposure, personal risk factors, COVID-19-like symptoms, needs of support during quarantine and sick leave was activated, if needed and were collected through the “Contacts epidemiological interview” included in the COVID-19 protocol [21]. CC were monitored on days 7 and 14 of quarantine and when any relevant symptoms were detected (fever, cough, or other COVID-19-like symptoms), a PCR test for COVID-19 was performed. Starting on August 7, the protocol was updated, and PCR was performed on all CC regardless of symptoms.

Strict General Data Protection Regulation compliance was ensured in regard to the database storage system and the contact tracing workforce granted accesses.

### Data sources and assessed Key Performance Indicators (KPI) and variables

As part of the monitoring and evaluation of the CT program, weekly KPI were collected, analysed and reported to inform and guide the region’s TTSI strategy.

Table 1 shows an overview, as well as its calculation, targets and rationale, of the four KPI that encompass 12 sub-indicators monitoring and evaluating the Catalan contact tracing program. The four designated study areas assessed the percentage of new SARS-CoV-2 cases in which CC are identified and reported to MCC (KPI1), the average number of CC per case and its associated exposure characteristics (KPI2), the percentage of identified CC traced and quarantined (KPI3) and their outcomes throughout follow-up in terms of symptom development and progression to case (KPI4).

**Table 1:** Key Performance Indicators (KPI) framework evaluating the Catalan Contact Tracing (CT) program

Indicator	Sub-indicator	Calculation	Target	Definition and rationale
**1.** Percentage of **cases with identified CC**	**1.1.** Number of confirmed cases	KPI1 is obtained from the division of 1.2 by 1.1, which are both direct outputs from MCC extractions. Results are stratified geographically and temporally	80%	Output indicator reflecting the system’s capacity to conduct case investigation and contact elicitation. The higher it is, the greater impact can the CT program have.
	**1.2.** Number of confirmed cases with identified CC			
**2.** Average number of **CC per case**	**2.1.** Number of identified CC	KPI2 is obtained from the division of 2.1 by 1.2. Both 2.1, 2.2 and 2.3 are direct outputs from MCC. Results are stratified by age, geo-temporally and setting of exposure	5	Output indicator translating both the quality and quantity of contact elicitation. Its disaggregation by setting of exposure informs other COVID-19 control measures, as well as facilitates index cases prioritization in case of high ratios of contacts per case, with the goal to prioritize high risk settings and timely detect potential clusters, trace CCs and avoid uncontrolled community transmission.
	**2.2.** Median and interquartile range			
	**2.3.** Minimum and maximum			
**3.** Percentage of **CC traced and quarantined**	**3.1.** Number of traced CC (“verified CC”)	KPI3 is obtained from the division of 3.2 by 3.1a. Both 3.1 (including a/b), 3.2 and 3.3 are direct outputs from MCC. Results are stratified geo-temporally	70%	Outcome indicator rendering the quality of contact tracers’ duty. Two aspects are assessed: 1. contact notification, quarantine instructions recommendation, social support needs identification and COVID-19-like symptoms investigation over tracing call at day 0 (through KPI 3.1–2); 2. contact adherence to quarantine during follow-up calls at day 7 and 14 (through KPI 3.3).
	**3.1a.** CC verified accepting follow-up			
	**3.1b.** CC verified to be excluded from follow-up			
	**3.2.** Number of verified CC accepting quarantine and follow-up			
	**3.3.** Total amount of follow-up calls performed (day 0, 7 and 14)			
**4.** Percentage of **new cases that were a known CC**	**4.1.** Number of followed-up CC developing COVID-19 symptoms at tracing call	4.1 is a direct output from MCC, 4.2 being calculated from the division of 4.1 by 3.2. 4.3 is a direct output from T19 platform, 4.4 being calculated from the division of 4.3 by the number of confirmed cases in T19 within the period of study. Results are stratified geo-temporally	80% with sustained and gradual increase over time	Outcome indicator elucidating the control over transmission chains. The more suspected cases identified early, as well as new cases reporting having been exposed to a known case, the greater the traceability and prevention of transmission in the community. KPI4 is an indirect measure of the secondary attack rate in our setting at the moment of the study
	**4.2.** Percentage of contacts with symptoms at tracing call			
	**4.3.** Number of new cases that were a known CC			

## Results

From 20 May to 31 December, 2020, a total of 301,522 new cases of SARS-CoV-2 infection (KPI 1.1) and its associated 923,072 CC (KPI 2.1) were registered in the MCC platform. A total of 202,451 cases had informed contacts.

### KPI 1. Percentage of cases with identified contacts

**Fig. 2:**
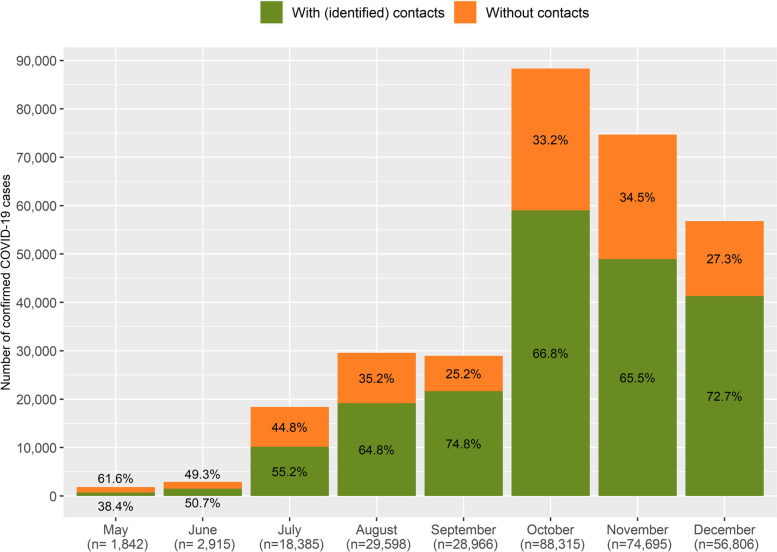
Number and percentage of confirmed COVID-19 cases with identified close contacts (KPI1). Catalonia, May–December 2020. The total COVID-19 cases reported is indicated under brackets in the lower side of the figure

The average percentage of informed cases over the study period was 67.1% (KPI1), with a significant increase from 38.4% in May to 72.7% in December (*p* < 0.05) (Fig. 2). Significant regional differences were observed (Table S2 and Fig. S1, *p* < 0.05).

### KPI 2. Average number of close contact per case

**Fig. 3:**
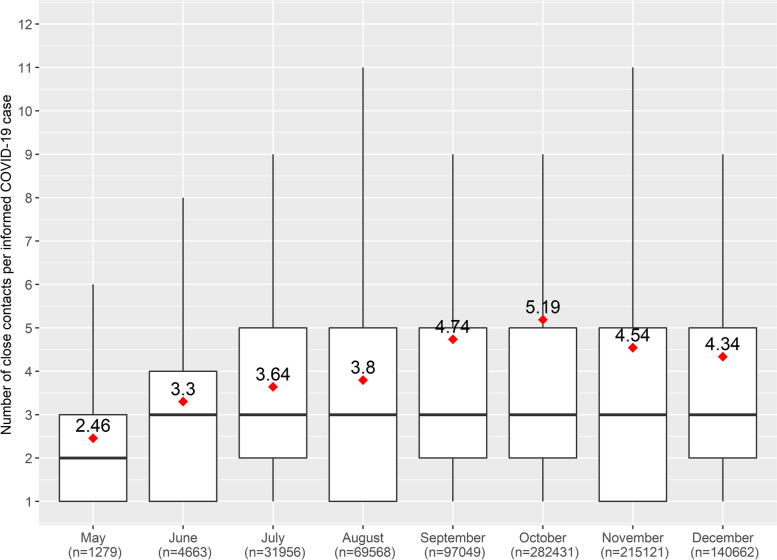
Number of close contacts per informed COVID-19 case (KPI2). Catalonia, May–December 2020. The monthly average number of contacts per informed COVID-19 case is incorporated inside the box

The average number of close contact per informed case was 4.6 (KPI2) during the study period, with a median of 3, an interquartile range 1–5 (KPI2.2) and a range of 1–243 (KPI2.3). The average per informed case experienced a sustained increase from 2.46 to 4.34 over time despite the staggering workload secondary to an escalation of cases (*p* < 0.05) (Fig. 3, Table S2). Notably 79.5% of the informed cases reported between 1 and 5 CC per case, and 13.1% reported between 6 and 10 CC (Table 2).

**Table 2:** Range of close contacts per informed COVID-19 case. Catalonia, May–December 2020

Number of close contacts per informed case	Informed cases	% of informed cases
1–5	160,867	79.5%
6–10	26,508	13.1%
11–15	4642	2.3%
> 15	10,434	5.2%
**Total**	**202,451**	**100,0%**

**Fig. 4:**
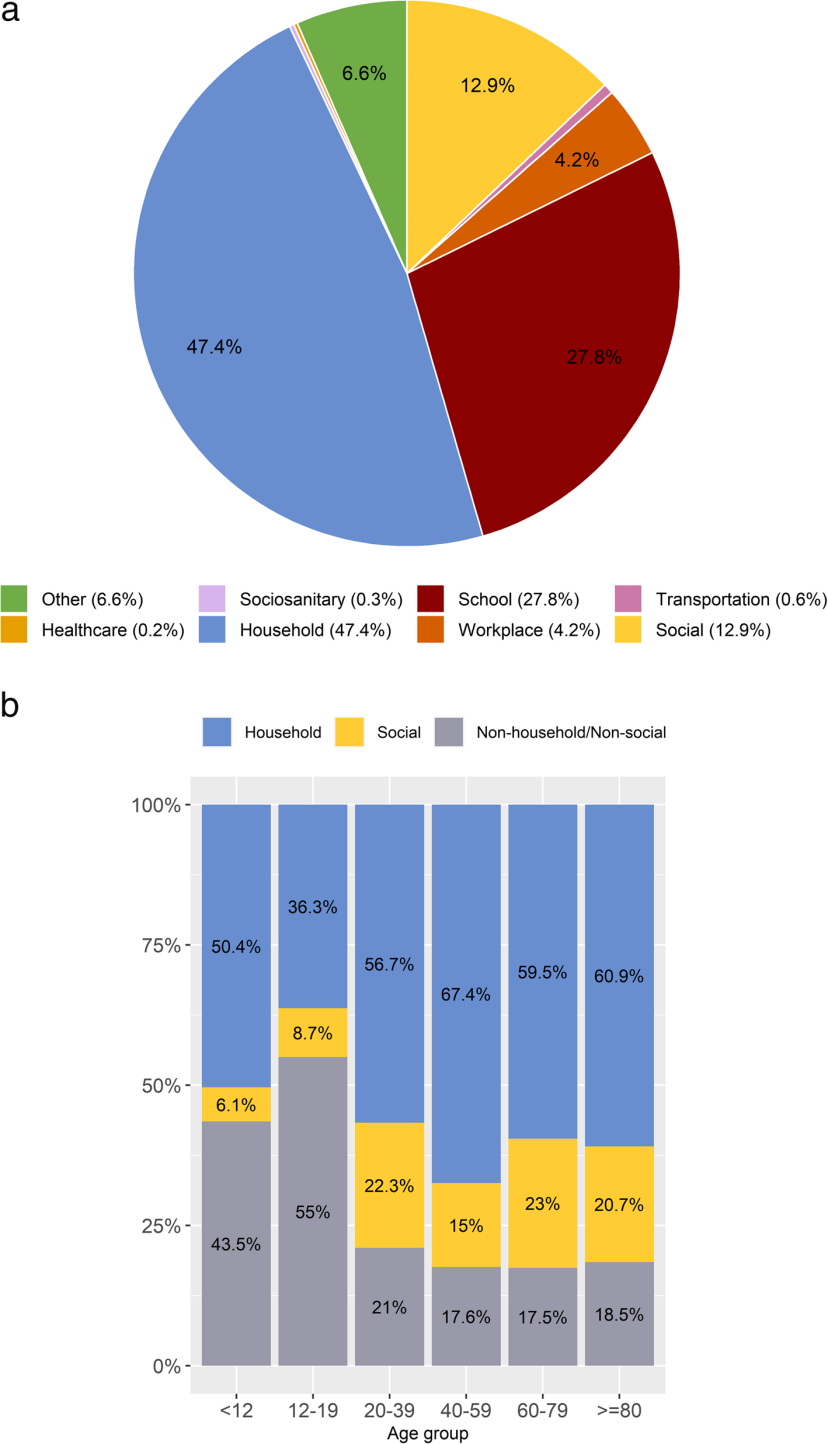
**a**: Distribution of close contacts stratified by setting exposure (Catalonia, data from August 2020). **b**. Distribution of close contacts by setting of exposure and age group (data from August 2020, Catalonia)

From August onwards, the setting of exposure in which the contact took place was systematically collected. The distribution of the CC registered from August to December (804,831 CC representing a 95.5% of all CC), displayed a clear predominance of household contacts (47.4%) versus other settings of exposure (Fig. 4a). Moreover, when we conducted an analysis with those CC reporting information on age (subset of 465,856, 57.9% of CC from August to December), we showed this metric was age-dependent (*p* < 0.05), with household being especially common among CC aged 40–59 (67.4%), while social setting peaks at age 20–39 years and 60–79 years (22.3 and 23.0%) (Fig. 4b).

### KPI 3. Percentage of close contact traced and quarantined

**Fig. 5:**
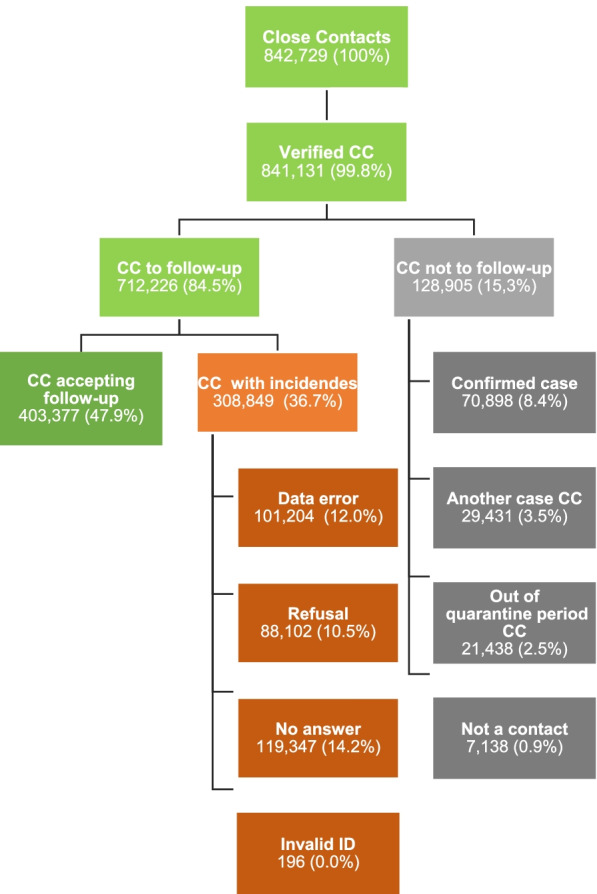
Flow chart elucidating the close contact verification process by tracers in Catalonia

From all identified CC, contact tracers verified 99.8% (841,131): 84.5% (712,226) were eligible to follow-up (KPI3.1a) and 15.3% (128,905) were excluded from the system due to four possible reasons (confirmed case, contact of another case, out of quarantine period or not a contact) (KPI3.1b). From the eligible CC to be followed-up, 47.9% (403,377) accepted and 36.7% (308,849) presented four type of incidences (data error, refusal, unanswered, invalid ID) which prevented contact tracers from following them up (Fig. 5 and Table S3).

**Fig. 6:**
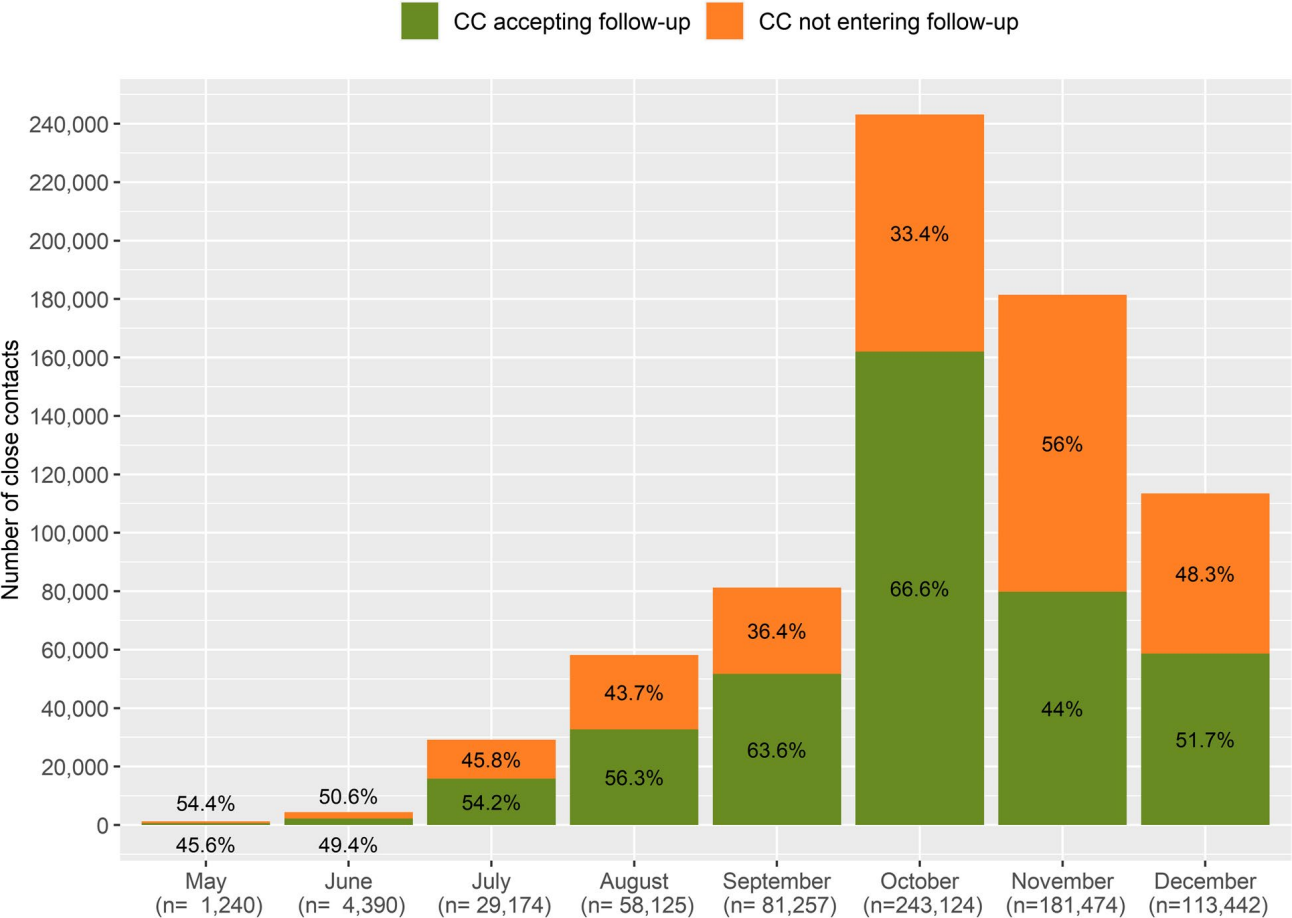
Number and percentage of close contacts traced and quarantined (KPI3). Catalonia, May–December 2020. The total amount of close contacts deemed necessary to follow-up (KPI3.1a) is indicated under brackets in the lower side of the figure

With an average of 56.6%, the percentage of traced CC accepting quarantine follow-up (KPI3) significantly improved from 45.6% in May to 66.6% in October, and descending to 51.7% in December due to the third wave registered in Catalonia (*p* < 0.05) (Fig. 6, Table S2). The nature of events preventing contact tracers from reaching out CC, significantly decreased the unanswered calls and improvement in data quality, in detriment of a greater refusal by CC (*p* < 0.05) (Table S3). The total follow-up calls performed at days 0, 7 and 14 throughout the study period was 970,067 (KPI3.3, Table S1).

### KPI 4. Percentage of new cases that were known close contact

A total of 32.672 CC (8.1% of CC accepting follow-up over the initial call) reported symptoms consistent with COVID-19 and, therefore, became suspected cases (KPI4.1–2). Significant differences were observed over the study period (Table S1).

**Fig. 7:**
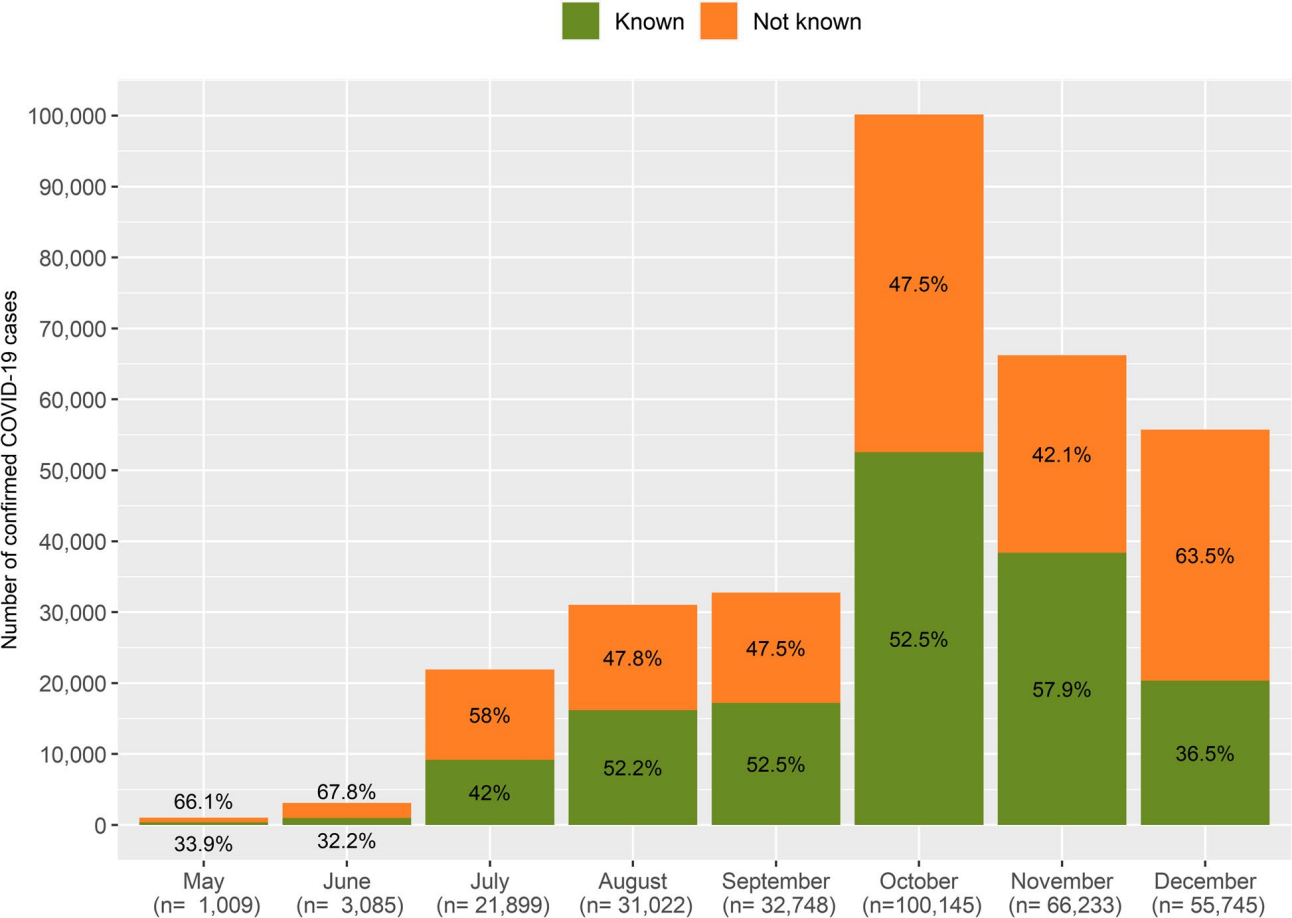
Number and percentage of new COVID-19 cases that were a known close contact (KPI4). Catalonia, May–December 2020. The total amount of new COVID-19 cases is indicated under brackets in the lower side of the figure

With an overall 53.3% in the percentage of new cases that had a known previous exposure to a confirmed case (KPI4, Table S2), this indicator related to the known transmission chains significantly increased from 33.9% in May to 57.9% in November decreasing again to 36.5% in December due to the third wave (*p* < 0.05) (Fig. 7, Table S2).

A summary of all KPI shown from May to December, 2020 can be found in the Table S1.

## Discussion

To our knowledge, this study is the first to present a systematic KPI framework to assess contact tracing performance with real country data, starting as soon as the contact tracing program was designed and progressively implemented. As the main agencies providing COVID-19 guidance updated their contact tracing recommendations [4, 26], our study provides with an opportunity to critically assess our evaluation system. As the Centers for Disease Control and Prevention highlights, the use and assessment of key indicators in contact tracing programs for COVID-19 is important as it allows to detect areas of improvement and implement changes to strengthen the program.

Despite the increase in new SARS-CoV-2 cases during our study period (especially May to October 2020, before the start of the third wave), all four KPI included in our contact tracing program raised significantly over time closely approaching the set targets. Our targets were set in a manner that contact tracing would play a key role to pandemic control, explaining its ambitious objectives. For instance, related to KPI1 (percentage of cases with identified contacts), its goal was established at 80% instead of 75% as Harvard TTSI strategy proposes [2]. Localized clusters of cases and outbreaks can put pressure in the limited human resources and therefore reduce the system’s capacity to timely finalize case investigation and identify contacts, as we observed, for instance, in Lleida during July and August 2020, a region that suffered from severe COVID-19 outbreaks explaining its low KPI1 values (Table S2). Moreover, our targets were rationalized according to socio-demographic characteristics in Catalonia. For example, Laxminarayan et al. [27] described an average of 7.3 CC per case in two Indian states (KPI2), whilst in our context 4.6 was the average over the study period, close to other European settings, like 2.4 CC per case in Italy [28].

The usefulness of stratifying results by household, social and non-household/non-social contacts’ setting of exposure is similar with other country strategies like the United Kingdom [29, 30]. The household setting predominance in our study is probably related to the nature of COVID-19 imposed restrictions upon mobility and social distancing. The household setting, more predominant among the CC aged 40–59, could be explained by habits from Spanish population and teleworking increasing since the pandemic. The social setting was more common among the CC aged 20 to 29 years, age group prone to outdoors activities and gatherings [31]. This finding exemplifies the need to tailor communication strategies to target audiences, as evidence suggests that response’s effectiveness partly depends on how communities receive, perceive, and act on the information provided by governments and other agencies [32]. Furthermore, KPI2 proved to be very practical in identifying potential superspreading events and contributed to early cluster investigation and detection of high-risk contacts, towards which epidemiologist can take additional control actions, such as urgent massive population screening [33].

In KPI3, we assessed the percentage of new contacts traced and quarantined by the contact tracers, identifying the need for governmental support (regarding social or housing support for quarantine compliance). After summer relaxation of restrictions, the increase in the number of cases during July till October (from 607 cases/day in July, to 2943 cases/day in October) put the system under pressure, but the system responded with increase in interviewers. KPI3 improved reaching the expected target of 70% (72.7%) in December (Table S1).

Regarding KPI4, the proportion of CC developing symptoms of COVID-19 during May–July was 10.7% average compared with August–December 8.2%, as during May–July, only symptomatic cases were tested and their CC interviewed. To assess SARS-CoV-2 secondary attack rate, we monitored the variable collected over case investigation on whether new cases are arising among contacts previously identified by the program. We observed a significant increase over time of this indicator (from 33.9% in May to 57.9% in November), translating the program’s effectiveness in interrupting SARS-CoV-2 transmission chains [4]. Universal CC testing established in early August, probably contributed to improve this indicator, contributing to detect asymptomatic cases.

The speed of testing, case investigation, and contact elicitation and tracing is essential for success in interrupting community transmission. Given that completeness data is extensively covered by KPI framework used in our program, in order to further improve the CT monitoring we propose expanding it with timeliness related KPIs as its shown in Table 3 adapted from international authors guidance [26, 34]. The new framework proposes a set of new process indicators prioritized according to the incidence of cases. After this study, we reviewed the indicators used in 2020 and during 2021 new indicator’s framework was proposed to be added (Table 3). During a high incidence period (> 3000 cases/day): “time from case symptoms onset to diagnosis”; “time from case diagnosis to interview and contact quarantine”; “CC per case disaggregate by risk category and settings” and “% of CC confirmed as new SARS-CoV-2 infection”. In a “low – moderate risk transmission” time, additional to main KPIs and the new previous indicators, four additional are proposed: percentage of cases with no CC to declare”; “percentage of tested CC for SARS-CoV-2”; “percentage of traced CC adherent to quarantine measures” and “percentage of vaccinated CC”, vaccination in Spain started on 27 December 2020.

**Table 3:** Contact tracing monitoring and evaluation through a proposal framework of Key Performance Indicators (KPIs)

**High risk transmission** ***Mitigation strategy***	**Low-moderate risk transmission** ***Maintenance - suppression strategy***
Incidence > 3000 cases/day (> 40 cases/100,000 inhab./day)	Incidence < 3000 cases/day (< 15/30 cases/100,000 inhab./day)
**Minimal set of CT KPIs (daily). Additional to the KPI1,2,3,4.**	**All previous CT KPIs plus the following (daily)**
1. Time from case symptoms onset to diagnosis 2. Time from case diagnosis to case interview and contact quarantine 3. CC per case (median, IQR, minimum, maximum), disaggregate by risk category and settings 4. % of CC confirmed as new SARS-CoV-2 infection	1. % of cases with no CC to declare 2. % of tested CC for SARS-CoV-2 3. % of traced CC adherent to quarantine measures 4. % of vaccinated CC
Prioritize **backward CT** and **test only high-risk CC** (cluster scoring), prioritise CC interview and follow up if < 7 days from last exposure to the case.	Set up **forward CT** and initiate **universal CC testing** (additionally to all previous)

Legend: percentage (%), informed case (IC), close contact (CC), inhabitants (inhab.), interquartil range (IQR), key performance indicator (KPI), contact tracing (CT)

Disaggregate KPI results by the following variables: geographically (regional epidemiological surveillance unit), sociodemographic (gender and age), exposure setting, CC symptom onset, type of COVID-19-like symptom, tracing incidence, contact media, vaccination status, day of follow-up

According to Kretzschmar et al. and other international public health agencies [35] CT will only be effective if the time from case symptom onset to reception of test results is within 3 days, and the time from receiving test results to quarantine of contacts is less than 1 day. In addition, we suggest two scenarios in KPI evaluation depending on the weekly SARS-CoV-2 incidence.

Measuring process indicators belonging to the “time from case symptom onset to diagnosis, and to case interview and contact quarantine” will allow identifying bottlenecks in the system and carrying out tailored measures in each geographical area depending on the area in which the delay is identified. Due to our two separated COVID-19 databases, storing either case or CC information has impeded its calculation, but at the time of writing this manuscript, it has been a fusion of the two databases and these metrics have been incorporated in our framework.

Centralizing contact management through a unique platform allowing diligent KPI appraisal is fundamental [25]. A special CT monitoring and evaluation team was set in the PHAC, in charge of assessing KPI on a daily basis and issuing weekly reports with overtime trends. A traffic light code was established for the four presented indicators and, under pre-defined thresholds, the concerned territories were closely followed-up in order to adjust the control strategy, either regionally or nationally.

Moreover, the scale-up of CT workforce with continuous training and support, as well as its integration with the primary care providers and the PHAC, has been a key cornerstone of the program. Towards the recommended minimum of 30 tracers per 100 k population, during emergencies [36], our workforce has been gradually heightened at every level (primary care, hospital facilities and epidemiological surveillance units).

Several limitations related to information systems hinder the systematic evaluation of CT and KPI in our context. First, the existence of de-centralized data systems for some occupational settings and nursery homes, mean these subsets of exposures are partially connected to the MCC platform and therefore underrepresented in our study. Second, the lack of integration of digital CT to complement manual efforts, as Catalonia has only systematically implemented a mobile application to follow-up confirmed cases’ symptoms (App STOP COVID19 CAT). Finally, in order to ensure “General Data Protection Regulation” compliance, no information that could potentially identify the index case was provided to the contact over initial notification call. Such legal constraint implied the date of last exposure, from which the 14 days quarantine was calculated, could not be disclosed [36], which haltered the estimation of quarantine adherence.

## Conclusions

As the COVID-19 pandemic unfolds, CT remains fundamental to support a TTSI strategy complimentary to vaccination rollout. The presented results highlight the success of attentive program monitoring and evaluation: despite not reaching the pursued targets, the consistent upward trend reflects the work performed on the system’s actionable incidences. The critical revision of the KPI framework aims to provide essential information in COVID-19 control policies. Adding timeliness indicators to identify delays and bottle-necks in the system will inform targeted interventions. This information has multiple public health implications since it will contribute to interrupt the spread of the SARS-CoV-2, to break chains of transmission in the community as well as to being prepared in front of other future epidemics. This work provides additional information on COVID-19 indicators framework experience from country’s real data, and allowed us to improve our monitoring tools in 2021–2022.

## Supplementary Information

**Additional file 1.**  

## Data Availability

All data relevant to the study are included in the article or uploaded as supplementary information. The datasets analyzed during the study are not publicly available (as they are personals patient’s data) but are available from the corresponding author on reasonable request.

## Abbreviations

COVID-19: Coronavirus disease
CT: Contact tracing
CC: Close contact
KPI: Key performance indicators
MCC: MediatorCovidContacts
PCR: Polymerase chain reaction
PHAC: Public Health Agency of Catalonia
SARS-CoV-2: Severe acute respiratory syndrome coronavirus 2
TTSI: T, diagnostic testing, T, contact tracing, and SI, supported isolation
XVEC: Epidemiological Surveillance Network of Catalonia
