# Supplementary material for: SARS-CoV-2 Catalonia contact tracing program: evaluation of key performance indicators
Source: BMC Public Health. 2022 Jul 20;22:1397. doi: 10.1186/s12889-022-13695-8 (PMC9299963; doi:10.1186/s12889-022-13695-8)
Supplement: Supplementary file 1 — Additional file 1. [file 12889_2022_13695_MOESM1_ESM.docx]

**SARS-CoV-2 Catalonia Contact Tracing Program: evaluation of Key Performance Indicators**

**SUPPLEMENTARY MATERIAL**

[Table S1: Summary table of the Key Performance Indicators of the Contact Tracing program. Catalonia, May-December 2020 3](#_Toc104375362)

[Table S2: Temporal evolution of the percentage of cases with identified close contacts (KPI1) by region. Catalonia, May-December 2020 5](#_Toc104375363)

[Figure S1: Temporal evolution of the percentage of cases with identified close contacts (KPI1) by region. Catalonia, May-December 2020 7](#_Toc104375364)

[Table 3: Descriptive analysis of main incidences preventing contact tracing (KPI3). Catalonia, May-December 2020 8](#_Toc104375365)

Table S1. Summary table of the Key Performance Indicators of the Contact Tracing program. Catalonia, May-December 2020

| **Key Indicator** | **Sub-indicator** | **May** | **June** | **July** | **August** | **September** | **October** | **November** | **December** | **Total** |
| --- | --- | --- | --- | --- | --- | --- | --- | --- | --- | --- |
| **1.** Percentage of **cases with identified CC** | **1.1.** Number of confirmed cases. | 1,842 | 2,915 | 18,385 | 29,598 | 28,966 | 88,315 | 74,695 | 56,806 | 301,522 |
|  | **1.2.** Number of confirmed cases with CCs identified and reported to MCC. | 707 | 1,477 | 10,153 | 19,172 | 21,653 | 59,031 | 48,946 | 41,312 | 202,451 |
|  | **1. Percentage of cases with identified CC.** | **38.4%** | **50,7%** | **55,2%** | **64,8%** | **74,8%** | **66,8%** | **65,5%** | **72,7%** | **67.1%^a^** |
| **2.** Average number of **CC per case** | **2.1.** Number of identified CCs. | 1,279 | 4,663 | 31,956 | 69,568 | 97,049 | 282,431 | 215,121 | 140,662 | 842,729 |
|  | **2.2.** Median and interquartile range. | 2 (1-3) | 3 (1-4) | 3 (2-5) | 3 (1-5) | 3 (2-5) | 3 (2-5) | 3 (1-5) | 3 (2-5) | 3 (2-5) |
|  | **2.3.** Minimum and maximum. | (1, 16) | (1, 32) | (1, 79) | (1, 112) | (1, 102) | (1, 243) | (1, 124) | (1, 77) | (1, 243) |
|  | **2. Average number of CC per case.** | **2.5** | **3.3** | **3.6** | **3.8** | **4.7** | **5.2** | **4.6** | **4.3** | **4.6^b^** |
| **3.** Percentage of **CC traced and quarantined** | **3.1.** Number of traced CC (“verified CC”). | 1,279 | 4,661 | 31,951 | 69,464 | 97,018 | 282,311 | 214,959 | 139,488 | 841,131 |
|  | **3.1a.** CC verified to be followed-up. | 1,240 | 4,390 | 29,174 | 58,125 | 81,257 | 243,124 | 181,474 | 113,442 | 712,226 |
|  | **3.1b.** CC verified to be excluded. | 39 | 271 | 2,777 | 11,339 | 15,761 | 39,187 | 33,485 | 26,046 | 128,905 |
|  | **3.2.** Number of verified CC accepting follow-up. | 566 | 2,167 | 15,812 | 32,727 | 51,673 | 161,980 | 79,797 | 58,655 | 403,377 |
|  | **3.3.** Total amount of follow-up calls performed (day 0, 7 and 14). | 1,491 | 4,754 | 31,453 | 76,083 | 135,232 | 422,670 | 140,439 | 157,945 | 970,067 |
|  | **3. Percentage of traced CC accepting quarantine follow-up.** | **45.6%** | **49.4%** | **54.2%** | **56.3%** | **63.6%** | **66.6%** | **44.0%** | **51.7%** | **56.6%^c^** |
| **4.** Percentage of **new cases that were known CC** | **4.1.** Number of followed-up CC developing COVID-19 symptoms. | 64 | 198 | 1,834 | 3,148 | 4,142 | 12,503 | 5,826 | 4,957 | 32,672 |
|  | **4.2.** Percentage of followed-up CC developing COVID-19 symptoms. | 11,3% | 9,1% | 11,6% | 9,6% | 8,0% | 7,7% | 7,3% | 8,5% | 8.1%^d^ |
|  | **4.3.** Number of new cases that were known CC. | 342 | 994 | 9,196 | 16,190 | 17,208 | 52,546 | 38,374 | 20,342 | 155,192 |
|  | **4. Percentage of new cases that were known CC.** | **33.9%** | **32.2%** | **42.0%** | **52.2%** | **52.5%** | **52.5%** | **57.9%** | **36.5%** | **53.3%^e^** |

^a^ p-value for the comparison of information on identified contacts by month: <0.05

^b^ p-value for the comparison of number of close contact per informed COVID-19 case by month: <0.05

^c^ p-value for the comparison of information on acceptance of follow-up by month: <0.05

^d^ p-value for the comparison of information on development of symptoms at the first call by month: <0.05

^e^ p-value for the comparison of cases with information on previous exposure to a confirmed case by month: <0.0

Table S2: Temporal evolution of the percentage of cases with identified close contacts (KPI1) by region. Catalonia, May-December 2020

| **Regions** | **MAY** | | |  | **JUNE** | | |  | **JULY** | | |  | **AUGUST** | | |  | **SEPTEMBER** | | |
| --- | --- | --- | --- | --- | --- | --- | --- | --- | --- | --- | --- | --- | --- | --- | --- | --- | --- | --- | --- |
|  | **Total cases** | **Cases with (identified) contacts** | **% cases with (identified) contacts** |  | **Total cases** | **Cases with (identified) contacts** | **% cases with (identified) contacts** |  | **Total cases** | **Cases with (identified) contacts** | **% cases with (identified) contacts** |  | **Total cases** | **Cases with (identified) contacts** | **% cases with (identified) contacts** |  | **Total cases** | **Cases with (identified) contacts** | **% cases with (identified) contacts** |
| Barcelona ciutat | 543 | 171 | 31,5% |  | 676 | 264 | 39,1% |  | 4,666 | 2,539 | 54,4% |  | 6,911 | 5,430 | 78,6% |  | 6,400 | 5,331 | 83,3% |
| Barcelona Sud | 355 | 123 | 34,6% |  | 421 | 209 | 49,6% |  | 4,399 | 2,445 | 55,6% |  | 6,035 | 3,533 | 58,5% |  | 4,817 | 3,555 | 73,8% |
| Barcelonès Nord-Maresme | 233 | 82 | 35,2% |  | 352 | 148 | 42,0% |  | 1,667 | 1,051 | 63,0% |  | 3,976 | 2,075 | 52,2% |  | 3,678 | 2,517 | 68,4% |
| Catalunya Central | 164 | 81 | 49,4% |  | 346 | 187 | 54,0% |  | 436 | 374 | 85,8% |  | 1,041 | 864 | 83,0% |  | 1,878 | 1,498 | 79,8% |
| Girona | 225 | 88 | 39,1% |  | 309 | 186 | 60,2% |  | 928 | 744 | 80,2% |  | 1,695 | 1,249 | 73,7% |  | 3,012 | 2,157 | 71,6% |
| Lleida Alt Pirineu i Aran | 302 | 144 | 47,7% |  | 767 | 464 | 60,5% |  | 4,650 | 1,907 | 41,0% |  | 2,523 | 1,284 | 50,9% |  | 2,412 | 1,405 | 58,3% |
| Tarragona i Terres de l’Ebre | 20 | 18 | 90,0% |  | 45 | 19 | 42,2% |  | 592 | 448 | 75,7% |  | 2,240 | 1,766 | 78,8% |  | 2,438 | 2,015 | 82,6% |
| Vallès Oriental i Vallès Occidental |  |  |  |  |  |  |  |  | 1,047 | 645 | 61,6% |  | 5,177 | 2,971 | 57,4% |  | 4,331 | 3,175 | 73,3% |
| **Total** | **1,842** | **707** | **38,4%** |  | **2,916** | **1477** | **50,7%** |  | **18,385** | **10,153** | **55,2%** |  | **29,598** | **19,172** | **64,8%** |  | **28,966** | **21,653** | **74,8%** |

| **Regions** | **OCTOBER** | | |  | **NOVEMBER** | | |  | **DECEMBER** | | |  | **TOTAL** | | | |
| --- | --- | --- | --- | --- | --- | --- | --- | --- | --- | --- | --- | --- | --- | --- | --- | --- |
|  | **Total cases** | **Cases with (identified) contacts** | **% cases with (identified) contacts** |  | **Total cases** | **Cases with (identified) contacts** | **% cases with (identified) contacts** |  | **Total cases** | **Cases with (identified) contacts** | **% cases with (identified) contacts** |  | **Total cases** | **Cases with (identified) contacts** | **% cases with (identified) contacts** |  |
| Barcelona ciutat | 18,543 | 13,507 | 72,8% |  | 13,947 | 11,407 | 81,8% |  | 11,542 | 8,860 | 76,8% |  | **63,228** | **47,509** | **75,1%** |  |
| Barcelona Sud | 13,516 | 8,862 | 65,6% |  | 12,535 | 7,806 | 62,3% |  | 9,374 | 7,699 | 82,1% |  | **51,452** | **34,232** | **66,5%** |  |
| Barcelonès Nord-Maresme | 11,731 | 7,324 | 62,4% |  | 9,309 | 5,963 | 64,1% |  | 6,343 | 4,692 | 74,0% |  | **37,289** | **23,852** | **64,0%** |  |
| Catalunya Central | 6,360 | 4,744 | 74,6% |  | 5,816 | 3,929 | 67,6% |  | 5,075 | 3,812 | 75,1% |  | **21,116** | **15,489** | **73,4%** |  |
| Girona | 9,437 | 5,854 | 62,0% |  | 8,783 | 5,087 | 57,9% |  | 7,576 | 4,968 | 65,6% |  | **31,965** | **20,333** | **63,6%** |  |
| Lleida Alt Pirineu i Aran | 4,217 | 2,640 | 62,6% |  | 5,423 | 3,178 | 58,6% |  | 3,804 | 2,567 | 67,5% |  | **24,098** | **13,589** | **56,4%** |  |
| Tarragona i Terres de l’Ebre | 9,162 | 6,805 | 74,3% |  | 7,601 | 4,908 | 64,6% |  | 5,188 | 3,256 | 62,8% |  | **27,286** | **19,235** | **70,5%** |  |
| Vallès Oriental i Vallès Occidental | 15,349 | 9,295 | 60,6% |  | 11,281 | 6,668 | 59,1% |  | 7,904 | 5,458 | 69,1% |  | **45,089** | **28,212** | **62,6%** |  |
| **Total** | **88,315** | **59,031** | **66,8%** |  | **74,695** | **48,946** | **65,5%** |  | **56,806** | **41,312** | **72,7%** |  | **301,523** | **202,451** | **67,1%** |  |

* p-value for the comparison of information on identified contacts by region and month: <0.05

Figure S1: Temporal evolution of the percentage of cases with identified close contacts (KPI1) by region. Catalonia, May-December 2020


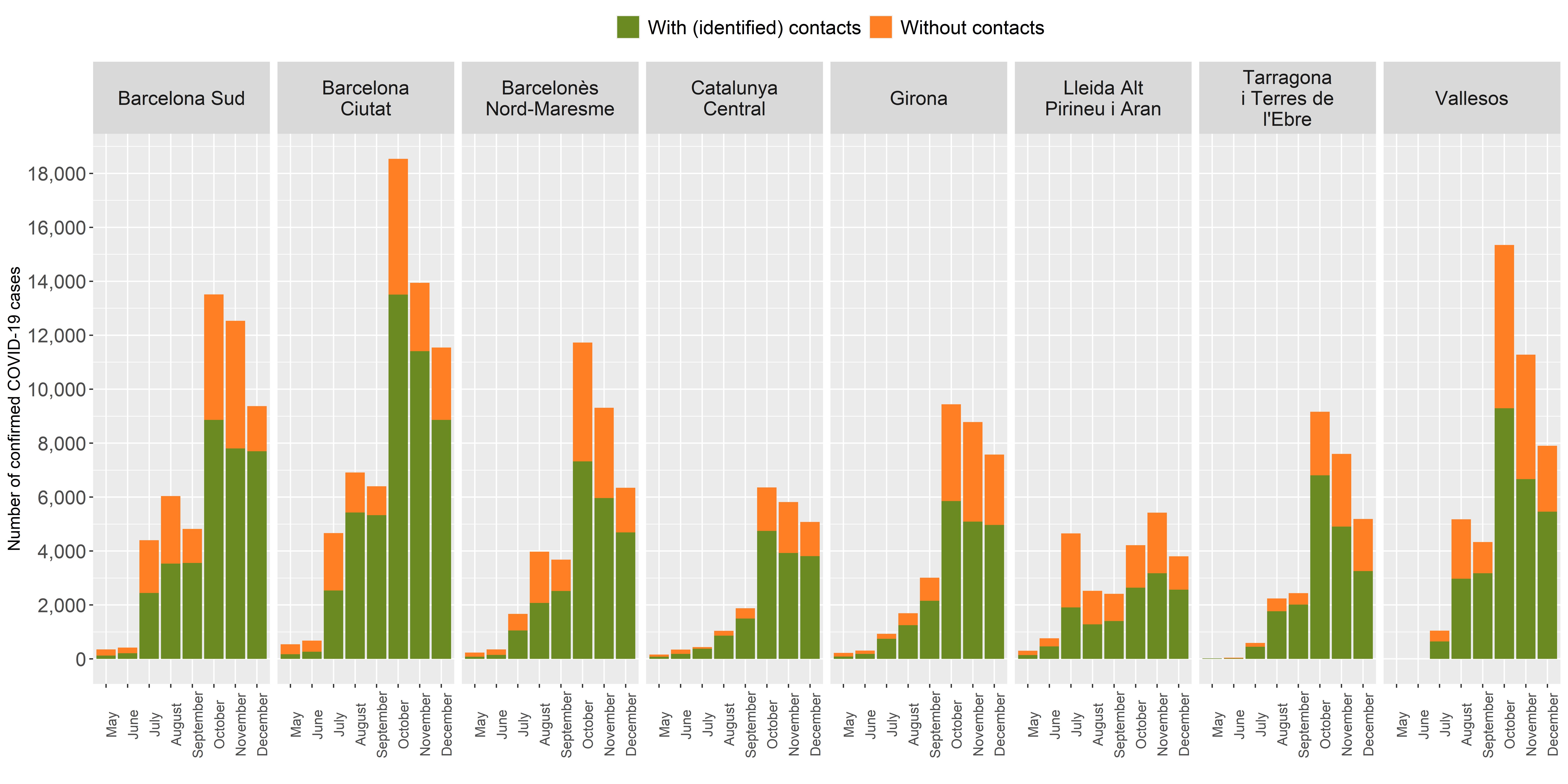


Table S3: Descriptive analysis of main incidences preventing contact tracing (KPI3). Catalonia, May-December 2020

|  | **Data error** | **Unanswered** | **Refusal** | **Invalid ID** | **Number of CC** | **p-value^a^** |
| --- | --- | --- | --- | --- | --- | --- |
| May | 32.60% | 16.89% | 3.13% | 0.08% | 1,279 | <0.05 |
| June | 27.33% | 14.03% | 6.26% | 0.06% | 4,661 |  |
| July | 30.97% | 4.58% | 6.22% | 0.04% | 31,951 |  |
| August | 17.46% | 5.93% | 13.16% | 0.01% | 69,464 |  |
| September | 12.65% | 5.42% | 12.39% | 0.04% | 97,018 |  |
| October | 12.28% | 5.47% | 10.99% | 0.00% | 282,311 |  |
| November | 8.04% | 28.76% | 10.47% | 0.03% | 214,959 |  |
| December | 9.50% | 21.78% | 7.95% | 0.04% | 139,488 |  |
| **Total** | **12.03%** | **14.19%** | **10.47%** | **0.02%** | **841,131** |  |

^a^ p-value for the comparison of the type of incidences during follow-up by month: <0.05
